# Antipsychotics and Seizure Risk in Patients with Brain Tumors: Mechanisms, Modifiers, and Clinical Insights

**DOI:** 10.1007/s11920-026-01696-y

**Published:** 2026-07-13

**Authors:** Fawad Taj, Herbert B. Newton, Yesne Alici

**Affiliations:** 1 Case Western Reserve University School of Medicine, Cleveland, OH 44106 USA; 2 Department of Psychiatry, Seidman Cancer Center, University Hospitals Cleveland Medical Center, 11100 Euclid Avenue, Cleveland, OH 44106 USA; 3 Neuro-Oncology Center and Brain Tumor Institute, University Hospitals of Cleveland Medical Center, Seidman Cancer Center, Hanna House 5th Floor, 11100 Euclid Avenue, Cleveland, OH 44106 USA; 4 Department of Psychiatry and Behavioral Sciences, Memorial Sloan Kettering Cancer Center, New York, NY USA

**Keywords:** Antipsychotics, Seizures, Epilepsy, Brain tumors, Neuro-oncology, Clozapine, Aripiprazole, Risperidone

## Abstract

**Purpose of Review:**

Neuropsychiatric symptoms including agitation, delirium, and psychosis are common in patients with primary or metastatic brain tumors and frequently necessitate antipsychotic treatment. Brain tumors create a hyperexcitable cortical environment characterized by glutamatergic excess, impaired inhibitory signaling, peritumoral edema, network disruption, and treatment-related neurotoxicity, all of which lower seizure threshold. Emerging data further implicates impaired astrocytic glutamate regulation, neuroligin-3–mediated neuron–glioma synaptic signaling, and IDH-mutant–associated oncometabolite accumulation as contributors to cortical instability. Neuropsychiatric symptoms often localize to frontal and temporal networks that are also highly epileptogenic, amplifying vulnerability. Brain tumor–related epilepsy further compounds this risk through recurrent seizures, interictal dysfunction, and antiseizure medication effects that contribute to cognitive and behavioral disturbance.

**Recent Findings:**

In general psychiatric populations, second-generation antipsychotics have low intrinsic seizure liability. However, these data largely exclude patients with structural brain disease. In neuro-oncology, seizure risk is context-dependent and reflects the interaction of tumor biology, antiseizure medication exposure, metabolic instability, and dynamic treatment effects. Clozapine and low-potency first-generation antipsychotics confer the greatest seizure risk, whereas risperidone and aripiprazole are associated with lower risk. Commonly used antiseizure medications, particularly levetiracetam, may exacerbate irritability and behavioral dysregulation, increasing the need for antipsychotic treatment within an already unstable neurophysiologic environment.

**Summary:**

This review synthesizes evidence on tumor-associated hyperexcitability, antipsychotic-specific seizure liability, and clinical modifiers of risk in brain tumor populations. We propose a seizure-informed, interdisciplinary framework that integrates tumor biology, antiseizure medication selection, and psychiatric indication to guide antipsychotic prescribing in neuro-oncology.

## Introduction

Neuropsychiatric symptoms are common in patients with primary or metastatic brain tumors frequently manifesting as agitation, delirium, and psychosis [1, 2]. These clinical presentations often necessitate the use of antipsychotic medications for symptom stabilization. Effective psychiatric stabilization is associated with improved cancer-related outcomes, reduced caregiver burden, and enhanced quality of life [3, 4].

Tumor-associated cortical hyperexcitability lowers the seizure threshold, and the use of proconvulsant medications such as antipsychotics further increases the risk. The Society for Neuro-Oncology (SNO) consensus review underscores the biological complexity of seizure production in brain tumor-related epilepsy, in which tumor-associated cortical hyperexcitability creates heightened vulnerability to seizure precipitation [5]. Peritumoral glutamate excess [6–8], impaired astrocytic reuptake [9], neuroligin-3–mediated neuron–glioma synapses [10], and IDH-mutant–associated D-2-hydroxyglutarate production [11] collectively create a hyperexcitable cortical environment in which even modest perturbations by pharmacologic agents may precipitate seizures.

Clinically, these mechanisms are associated with a high incidence of seizures across brain tumors, and is substantially higher in selected low-grade and glioneuronal tumors, rather than uniformly high across all tumors [5]. Susceptibility varies with tumor type, progression, edema, postoperative changes, and metabolic instability [12, 13].

Psychosis and behavioral disturbances are not unique to neuro-oncology, as other structural neurological disorders may also present with similar symptoms. Across these disorders, patients demonstrate heightened sensitivity to psychotropic medications, including increased risk of extrapyramidal symptoms, sedation, cognitive impairment, and delirium [2]. These vulnerabilities likely reflect a shared pathophysiologic substrate. Despite this, existing guidelines offer minimal guidance regarding the safe administration of antipsychotics, especially in neuro-oncology patients.

Brain tumor–related epilepsy (BTRE) contributes to neuropsychiatric and quality-of-life disturbances through network disruption, seizure burden, antiseizure medication effects, and psychological stress. The concept of “BTRE-induced disability” highlights this cumulative burden and underscores the need for individualized psychopharmacologic decision-making that integrates tumor biology, seizure risk, and psychiatric considerations [1].

Psychopharmacological decisions in brain-tumor patients therefore requires particular caution. Although seizure rates vary widely by tumor type, low-grade gliomas represent one of the highest-risk groups, with some series reporting seizures in up to 90% of adults; nearly half may remain refractory to first-line antiseizure monotherapy, often necessitating polytherapy [14]. Concurrently, commonly used antiseizure medications, such as levetiracetam, may contribute to psychiatric adverse effects; this is particularly evident when used prophylactically in seizure-naïve patients, where higher rates of agitation, anxiety, irritability, and mood symptoms have been observed compared with established BTRE [15]. In this context of high seizure burden and treatment complexity, the combined use of antiseizure medications and antipsychotics may further amplify seizure risk.

Although large-scale randomized data indicate that second-generation antipsychotics do not increase seizure risk in the general psychiatric population [16], these trials routinely exclude patients with structural brain injury, including brain tumors. This further highlights the evidence gap.

In this review, we synthesize current evidence on seizure epidemiology in brain tumors, examine the seizure risk associated with specific antipsychotic agents, and highlight tumor- and treatment-related modifiers of risk, with the aim of informing practical, collaborative neuro-oncologic care.

## Seizure Vulnerability in Brain Tumor Populations

### Epidemiology of Brain Tumor–Related Epilepsy (BTRE)

Seizures are a frequent complication of intracranial neoplasms and often constitute the initial clinical manifestation of disease. In many instances, the first seizure leads to tumor diagnosis [17]. Across broad brain tumor populations, more than one-third of patients develop epilepsy [17, 18]. However, Seizure prevalence varies widely by tumor type: dysembryoplastic neuroepithelial tumors (DNETs) and gangliogliomas demonstrate seizure rates of 80–100%, low-grade IDH mutant oligodendrogliomas and astrocytomas around 65–75%, meningiomas 30–60%, high-grade astrocytomas 30–50%, brain metastases 20–35%, and primary CNS lymphoma approximately 10% [17, 18].

Tumor location, type, and growth pattern are important determinants of seizure risk. Tumor type influences seizure risk through differences in growth rate, cortical involvement, neuronal/glioneuronal differentiation, peritumoral glutamate handling, edema, necrosis, hemorrhage, and molecular features such as IDH mutation. In a retrospective assessment of 124 primary brain tumors, high-grade gliomas in patients presenting with seizures tended to be smaller in size than tumors presenting with other neurological symptoms [19]. In contrast, low-grade gliomas in patients with seizures were larger and frequently located in the temporal lobe or the insula [19]. Although both low- and high-grade gliomas can be infiltrative, slow-growing cortically based tumors may allow prolonged remodeling of adjacent neuronal networks, whereas high-grade gliomas often contribute to seizures through necrosis, hemorrhage, edema, and acute disruption of inhibitory circuits [17, 18].

### Mechanisms of Tumor-Related Hyperexcitability

BTRE originates not within the tumor itself, which is electrically inert, but in the adjacent peritumoral cortex, where multiple interacting biological processes shift the excitatory–inhibitory balance toward hyperexcitability [17].

#### Glutamatergic Excess and Impaired Buffering

Peritumoral astrocytes exhibit reduced glutamine synthetase, impairing glutamate metabolism, and increasing extracellular glutamate concentrations that enhance neuronal excitation [17]. Infiltrating tumor cells may further disrupt glutamate homeostasis [18].

#### Disrupted Inhibition and Network Remodeling

Slow-growing glial and glioneuronal tumors promote cortical dysplasia, aberrant synaptic connectivity, and deafferentation, creating circuits highly susceptible to synchronous epileptiform activity [17]. “Secondary epileptogenesis,” characterized by epileptic foci remote from the tumor margin, has been well documented [17].

#### Microenvironmental and Metabolic Factors

Tumor infiltration and abnormal angiogenesis contribute to local hypoxia, acidosis, inflammation, oxidative stress, and gene expression changes, each capable of lowering the seizure threshold [17]. Blood–brain barrier disruption permits serum proteins and inflammatory mediators to enter cortical tissue, amplifying excitability.

#### Tumor-specific Influences

Low-grade gliomas appear to generate seizures primarily through chronic network distortion and excitatory plasticity [18]. High-grade gliomas provoke seizures through necrosis, hemorrhage, and edema, producing acute injury to inhibitory cortical circuits [17]. Neuronal tumors, including DNETs and gangliogliomas, contain dysplastic neurons that are intrinsically hyperexcitable [17].

Collectively, these mechanisms create a cortical milieu predisposed to seizure activity. In this setting, even small reductions in inhibitory signaling or increases in excitatory transmission, including those induced by antipsychotics, may have clinically significant consequences.

### Treatment-Related Modifiers of Seizure Threshold

Seizure susceptibility in brain tumor patients is dynamic and evolves over the course of oncologic treatment.

#### Surgery

Surgical resection is the most effective intervention for seizure control in low-grade gliomas. Complete resection is associated with markedly improved seizure outcomes [17, 18]. However, the early postoperative period is characterized by heightened seizure risk related to tissue manipulation, edema, and metabolic changes [17].

#### Radiotherapy

Radiation therapy reduces seizure frequency in 40–100% of patients with unresectable low-grade gliomas and can improve seizure control even before radiographic response [20]. However, radiation necrosis, delayed edema, and white-matter injury may provoke seizures later in the disease course [17].

#### Chemotherapy and Antiseizure medications (ASMs)

Chemotherapeutic agents such as temozolomide often reduce seizure burden in gliomas, consistent with antitumor activity [20]. Chemotherapy-related toxicity and metabolic disturbances, including corticosteroid-induced hyperglycemia and electrolyte abnormalities, may further modify seizure risk [17, 21]. Drug–drug interactions with antiseizure medications (ASMs) are also common: enzyme-inducing ASMs (phenytoin, carbamazepine) accelerate metabolism of irinotecan, etoposide, taxanes, tyrosine kinase inhibitors, and corticosteroids, potentially compromising antitumor efficacy and affecting seizure control [17]. For this reason, non–enzyme-inducing ASMs are generally preferred, including levetiracetam, lamotrigine, gabapentin, pregabalin, and lacosamide. Levetiracetam is widely used because it can be titrated rapidly and is available intravenously, though behavioral adverse effects occur in up to 12% of patients with brain tumors [17]. Lamotrigine requires slow titration due to the risk of rash. Gabapentin and pregabalin exhibit favorable interaction profiles but are supported primarily by retrospective series. Polytherapy may further increase adverse effects, and valproic acid warrants separate consideration because it can increase exposure to co-administered drugs, including lamotrigine. When antipsychotics are added, interactions may affect both tolerability and efficacy; enzyme-inducing ASMs can lower serum concentrations of some antipsychotics, potentially reducing effectiveness in the treatment of psychosis, while additive sedation and cognitive impairment may further complicate care [22–25].

#### Targeted Therapies

Targeted therapies have variable effect on seizure risks. Enzalutamide (androgen receptor inhibitor) shows increased seizure incidence in metastatic castration-resistant prostate cancer, likely through decreased GABA-gated chloride channels. EGFR inhibitors, including gefitinib, erlotinib, cetuximab, and panitumumab, may predispose to seizures through electrolyte depletion, particularly in patients with CNS involvement. Blinatumomab is associated with substantial neurologic toxicity, including [26].

#### Systemic and Metabolic Factors

Systemic and metabolic precipitants may further lower seizure threshold in patients with brain tumors. Common triggers, including hyponatremia, sleep deprivation, infection, steroid effects, hyperglycemia, and hypoglycemia, can acutely lower seizure threshold and are frequently encountered in neuro-oncology settings [17].

## Neuropsychiatric Burden in BTRE

### Global Neuropsychiatric Disturbances in CNS Tumors

Psychiatric and neurocognitive disturbances in brain tumor patients arise not only from tumor-related hyperexcitability but also from predictable patterns of network disruption related to lesion location. Frontal lobe tumors may produce disinhibition, apathy, impulsivity, personality change, and mood dysregulation; temporal lobe involvement may contribute to hallucinations, fear, paranoia, emotional instability, and psychosis; parietal lesions may cause misidentification syndromes, visuospatial distortions, or neglect; occipital tumors may trigger visual hallucinations, illusions, and palinopsia; and cerebellar pathology may lead to emotional lability, irritability, anxiety, or cognitive-affective syndromes. These manifestations often precede neurological signs, are easily misattributed to primary psychiatric illness, and profoundly affect quality of life [27].

Because many of the cortical regions most associated with these symptoms, particularly frontal and temporal networks, also carry substantial epileptogenic potential, psychopharmacologic management in this population should be individualized and seizure-informed [27].

### Neuropsychiatric Burden in BTRE and Overlap with Other Neurologic Diseases

Neuropsychiatric symptoms in patients with CNS tumors arise through at least two overlapping pathways: direct effects of tumor location, edema, mass effect, and oncologic treatment; and BTRE-related effects, including recurrent seizures, interictal dysfunction, ASM toxicity, and the psychological burden of epilepsy. The concept of “BTRE-induced disability” captures this cumulative impact on behavior, cognition, and quality of life and supports individualized psychopharmacologic decision-making that integrates seizure risk with psychiatric need [1].

Similar patterns are seen across other structural neurologic disorders, including stroke, traumatic brain injury, Parkinson disease, and dementias, where hallucinations, delusions, agitation, and related syndromes are associated with worse functional outcomes, greater caregiver burden, and increased mortality. These populations also demonstrate heightened sensitivity to psychotropic medications, including extrapyramidal symptoms, sedation, cognitive impairment, and delirium [2].

In BTRE, where tumor-related injury, recurrent seizures, and antiseizure medication burden intersect, antipsychotic regimens may be less well tolerated. This underscores the importance of an individualized, seizure-informed, cognition-aware, and interdisciplinary approach to psychopharmacologic management in brain tumor patients [1, 2, 27].

## Seizure Risk and Antipsychotic Medications

### General Seizure Liability of Antipsychotics

Psychotropic medications, including antipsychotics, interact with seizure susceptibility in ways that are agent-, dose-, and context-dependent, shaped by underlying neurobiology, comorbid psychiatric illness, and medication-specific pharmacology [28–31]. Historical concern regarding psychotropic-induced seizures has often been driven by case reports, overdose scenarios, and studies that did not adequately account for baseline seizure vulnerability. Contemporary evidence suggests that, at therapeutic doses, most second-generation antipsychotics do not carry substantial intrinsic epileptogenicity in the general psychiatric population; rather, seizure emergence more often reflects the interaction between medication exposure and a biologically vulnerable brain [16, 32, 33].

This distinction is particularly important in neuro-oncology. In the general population, the incidence of first unprovoked seizure is low, 0.07% to 0.09%, and randomized trial data suggest that second-generation antipsychotics are not associated with a significant increase in seizure risk compared with placebo, 0.05% to 0.15% [16, 32]. In a meta-analysis of 314 randomized placebo-controlled trials including 42,600 participants exposed to antipsychotics and 25,042 exposed to placebo, second-generation antipsychotics were not associated with increased seizure risk overall (37 vs. 28 seizures; 0.09% vs. 0.11%; pooled RR, 0.68 [95% CI, 0.41–1.12]) [16]. However, these data have limited direct applicability to patients with brain tumors because such trials routinely exclude individuals with structural brain lesions, epilepsy, or major neurologic comorbidity [32].

In patients with epilepsy or structural brain disease, antipsychotic-related seizure risk is better understood as the product of medication exposure superimposed on a destabilized neural network [24, 25, 29, 32, 34]. Clinical factors such as rapid titration, higher doses, abrupt withdrawal, polypharmacy, sleep deprivation, electrolyte disturbance, infection, and fluctuating metabolic state may further amplify this vulnerability [29, 32–34]. In patients with brain tumors, these general risk factors coexist with tumor-related hyperexcitability, antiseizure medication effects, and evolving oncologic treatment exposures, making extrapolation from general psychiatric cohorts inherently limited.

### Comparative Seizure-risk Hierarchy Across Antipsychotic Agents

Despite the limitations of available data, a relative hierarchy of seizure liability has emerged across antipsychotic agents, with seizure risk increasing at higher doses and with rapid titration [28, 29, 32, 33, 35, 36]. Clozapine consistently carries the greatest seizure risk, with observational data demonstrating a clear dose-response relationship and substantially higher incidence at doses above 600 mg/day or at elevated plasma concentrations [34, 35]. Among first-generation agents, low-potency antipsychotics such as chlorpromazine appear to confer greater risk than high-potency agents such as haloperidol and most most second-generation antipsychotics [29, 36].

Among second-generation antipsychotics, seizure risk is generally lowest with aripiprazole and risperidone, intermediate with olanzapine and quetiapine, and highest with clozapine [24, 29, 33, 35, 36]. Ziprasidone is also generally regarded as a relatively lower-risk second-generation option, although the evidence base is smaller and less specific to structural brain disease [33, 37]. EEG abnormalities occur in approximately 7% of treated patients, and clinical seizures in approximately 0.5% to 1% of those without preexisting epilepsy, though these estimates derive largely from general psychiatric populations rather than neurologically vulnerable cohorts [28, 29].

Comparative observational data support this gradient, but the absolute risk attributable to any single agent remains difficult to isolate from confounding by psychiatric severity, concomitant medications, dose escalation, and indication bias [30–32, 36]. Clozapine is a notable exception, for which seizure liability is sufficiently consistent across datasets to be regarded as clinically established [34, 35]. By contrast, the lower-risk designation of risperidone, aripiprazole, and ziprasidone should be interpreted as relative rather than absolute, particularly when these agents are used in patients with epilepsy, structural brain injury, or ongoing cortical instability [24, 25, 33].

Data remain limited for newer agents such as lurasidone, brexpiprazole, cariprazine, lumateperone, and pimavanserin, and their seizure liability is not yet well defined in patients with epilepsy or structural brain disease [38, 39]. Accordingly, in neuro-oncology practice, clinical decision-making still relies primarily on the better-characterized hierarchy established with older first-generation agents and more commonly used second-generation antipsychotics. See Table 1 for relative seizure risk across antipsychotic medications.

**Table 1:** Relative seizure liability of antipsychotics and practical considerations in BTRE

Antipsychotic	Relative seizure liability	Pharmacologic features relevant to seizure threshold	Parenteral formulation considerations	Major cautions in BTRE/neuro-oncology	Practical role
Clozapine [24, 29, 32–35, 37]	Highest [24, 29, 32–35]	Broad receptor-binding profile; substantial muscarinic, histaminergic, serotonergic, and adrenergic activity; clear dose dependence [29, 33, 35, 39]	No meaningful acute parenteral role [40, 41]	Strongest seizure concern; cumulative exposure matters; requires close co-management [34, 35]	Reserve for refractory cases only [24, 25, 33]
Chlorpromazine [29, 36, 37]	High [29, 36, 37]	Low-potency FGA with broad receptor effects and greater anticholinergic/sedative burden [29]	Historical parenteral use, but generally not preferred here [40]	Less attractive in BTRE given seizure and delirium burden [24, 29]	Avoid when lower-risk options are available [24, 29, 33]
Haloperidol [29, 36, 37]	Lower than low-potency FGAs [29, 36, 37]	High-potency D2 antagonism; relatively lower seizure liability than low-potency FGAs [29, 36, 37]	IM and IV use in hospital practice [40, 41]	QT prolongation, EPS, akathisia; IV use requires cardiac caution [40, 41]	Reasonable option for delirium or acute agitation when used conservatively [24, 29, 40, 41]
Risperidone [24, 25, 29, 32, 33, 36, 37]	Lower [24, 25, 29, 32, 33, 36]	Potent D2 and 5-HT2A antagonism with minimal muscarinic activity and narrower receptor burden [24, 29, 33, 39]	No short-acting intramuscular role emphasized here [40, 41]	Hypotension, EPS, prolactin-related effects at higher doses [24, 33]	Strong oral option when seizure liability is a major concern [24, 25, 33]
Aripiprazole [24, 25, 32, 33, 37]	Lower [24, 25, 32, 33, 37]	D2 partial agonism; 5-HT1A partial agonism; low muscarinic burden; less abrupt dopaminergic suppression [33, 37–39]	Not discussed here as a current short-acting U.S. inpatient option [40, 41]	Akathisia may complicate interpretation of agitation [24, 41]	Attractive oral option in seizure-vulnerable patients [24, 25, 33]
Olanzapine [24, 29, 32, 33, 36, 37]	Intermediate [24, 29, 32, 33, 36]	Broader multi-receptor profile; H1 and muscarinic activity; more sedating [29, 33, 39]	IM formulation available [40, 41]	More caution than risperidone/aripiprazole; watch oversedation and delirium layering [40, 41]	Useful in selected cases, but not the lowest-risk option [24, 33, 40, 41]
Quetiapine^ [24, 29, 32, 33, 36, 37]	Intermediate [24, 29, 32, 33, 36]	Diffuse receptor profile; strong H1 effects; relatively loose D2 binding [29, 33, 39]	No acute parenteral role [40, 41]	Sedation, orthostasis, delirium masking; intermediate seizure concern [24, 33]	Useful oral option when sedation is desired, but use cautiously [24, 25, 33]
Ziprasidone [24, 33, 37, 40, 41]	Lower [24, 33, 37]	Lower antimuscarinic burden than olanzapine/clozapine; generally lower seizure concern in comparative literature [24, 33, 37]	IM formulation available [40, 41]	QT prolongation remains important; evidence in structural brain disease is limited [33, 40, 41]	Reasonable IM option when lower seizure liability is desired [40, 41]
Lurasidone [38, 39]	Uncertain/limited data [38, 39]	Limited muscarinic activity; pharmacologically plausible lower risk, but evidence is sparse [38, 39]	No acute parenteral role	Limited BTRE-specific data [38]	Consider data-limited
Brexpiprazole [38, 39]	Uncertain/limited data [38, 39]	Partial agonist profile may be favorable, but evidence is limited [38, 39]	No acute parenteral role	Insufficient seizure-specific evidence [38, 39]	Consider data-limited
Cariprazine [38, 39]	Uncertain/limited data [38, 39]	D3/D2 partial agonist profile; limited seizure literature [38, 39]	No acute parenteral role	Insufficient seizure-specific evidence [38, 39]	Consider data-limited
Lumateperone [38, 39]	Uncertain/limited data [38, 39]	Novel multimodal mechanism; little evidence in epilepsy or structural brain disease [38, 39]	No acute parenteral role	Evidence gap in BTRE [38, 39]	Consider data-limited
Pimavanserin [39]	Uncertain/limited data [39]	Non-dopaminergic 5-HT2A inverse agonist/antagonist; little direct seizure data in BTRE [39]	No acute parenteral role	Evidence gap in tumor-related epilepsy [39]	Niche use only

Abbreviations: BTRE, brain tumor-related epilepsy; D2, dopamine D2 receptor; EPS, extrapyramidal symptoms; FGA, first-generation antipsychotic; H1, histamine H1 receptor; IM, intramuscular; IV, intravenous; 5-HT, serotonin.

^1^Relative seizure-liability categories are based on systematic review, observational psychopharmacology data, epilepsy literature, and limited preclinical evidence rather than direct head-to-head studies in patients with brain tumors [16, 24, 25, 28–39].

^2^“Lower” does not mean seizure-neutral; risk remains context-dependent and may be magnified in structurally vulnerable brains [24, 25, 32, 33].

^3^Acute hospital formulation considerations refer to practical inpatient use and should not be interpreted as evidence that route of administration independently determines seizure risk [40, 41]

### Mechanistic Basis for Differences in Seizure Liability

The observed differences in seizure liability across antipsychotics likely reflect overall receptor-binding architecture rather than a single shared proconvulsant mechanism. This is especially relevant in patients with brain tumor-related epilepsy (BTRE), where baseline cortical excitability is already amplified [5, 17, 18, 21]. In that setting, the pharmacodynamic “fit” of a given antipsychotic may matter more than in the general psychiatric population. Clozapine remains the clearest example of an antipsychotic with substantial seizure liability. Its elevated risk appears to reflect not high-potency D2 antagonism alone, but rather a broad and diffuse receptor-binding profile, including relatively weak and transient D2 occupancy alongside prominent activity across serotonergic, muscarinic cholinergic, histaminergic, and adrenergic receptors [29, 33, 38, 39]. Clozapine’s strong antimuscarinic burden may disrupt cholinergic modulation of cortical circuits, while substantial H1 antagonism and broader sedative effects may further alter cortical synchrony in ways that lower seizure threshold [29, 33, 39]. The dose dependence of clozapine-related seizures supports the view that cumulative receptor burden and exposure, rather than one isolated receptor effect, underlie its clinical epileptogenicity [34, 35].

Olanzapine and quetiapine appear to occupy an intermediate position. Although substantially less epileptogenic than clozapine, both share elements of a broader multi-receptor profile, including greater H1 antagonism and more diffuse non-D2 binding than risperidone or aripiprazole [29, 33, 38, 39]. Olanzapine also has meaningful muscarinic receptor affinity, which may contribute to its less favorable seizure profile relative to risperidone [39]. Quetiapine, despite relatively loose and rapidly dissociating D2 binding, has prominent antihistaminergic properties and broad receptor activity that may become more relevant in an already unstable cortex [33, 39].

By contrast, risperidone appears to have a more favorable seizure profile in part because it combines potent D2 and 5-HT2A antagonism with minimal muscarinic activity and a comparatively narrower non-dopaminergic receptor burden [24, 29, 33, 39]. Aripiprazole is mechanistically distinct from both groups, acting as a D2 partial agonist rather than a full antagonist, with additional partial agonism at 5-HT1A receptors and functionally antagonistic effects at 5-HT2A receptors [33, 38, 39]. This pharmacodynamic profile has been described as dopaminergic stabilization and may reduce the degree of abrupt dopaminergic suppression and network disequilibrium associated with full D2 blockade [33]. It also carries negligible muscarinic activity and relatively modest H1 burden compared with clozapine, olanzapine, or quetiapine [38, 39].

Preclinical data are broadly consistent with this hierarchy. In a genetically epilepsy-prone rat model, clozapine showed the clearest proconvulsant signal, whereas risperidone and olanzapine worsened seizure expression primarily at higher exposures. Under the conditions studied, haloperidol, quetiapine, and aripiprazole did not significantly worsen seizure severity, and aripiprazole demonstrated anticonvulsant properties in that model [37]. Although animal data cannot be directly extrapolated to clinical prescribing, they reinforce the broader principle that antipsychotics do not affect epileptogenic circuitry uniformly and that receptor-level differences may be clinically meaningful when baseline cortical vulnerability is high.

Taken together, these data suggest that in BTRE, agents with minimal antimuscarinic activity, lower H1 burden, and less disruptive effects on dopaminergic signaling, particularly risperidone and aripiprazole, may be preferable when an antipsychotic is required [24, 25, 29, 33]. Clozapine should be used with particular caution, and olanzapine or quetiapine may warrant greater vigilance than their “atypical” designation alone might imply. In neuro-oncology, therefore, seizure risk should be understood not simply as a property of the drug, but as the interaction between drug-specific pharmacology and a tumor-bearing brain already close to seizure threshold.

### Parenteral and Intravenous Antipsychotic Use in Hospitalized Patients

In hospitalized patients with brain tumors, antipsychotics are often initiated not for chronic psychotic disorders but for acute agitation, delirium, severe behavioral dyscontrol, or inability to take oral medication. In these settings, intramuscular administration may be required to achieve rapid symptom control. This route of administration is particularly relevant in neuro-oncology, where postoperative delirium, steroid-related agitation, prolonged postictal confusion, metabolic encephalopathy, and fluctuating consciousness may create urgent indications for parenteral treatment [40, 41]. However, parenteral administration does not eliminate the seizure-risk principles that govern oral prescribing; rather, it compresses them into a shorter time frame in a medically unstable population [24, 29, 32, 33].

The available literature does not establish that intramuscular administration independently confers higher intrinsic seizure risk than oral use for most antipsychotics. Instead, seizure risk in these settings appears to remain primarily agent-, dose-, and patient-dependent, while being amplified by the circumstances in which parenteral agents are typically used: severe agitation, delirium, polypharmacy, infection, electrolyte disturbance, hypoxia, sleep deprivation, recent neurosurgery, corticosteroid exposure, and structural brain vulnerability [24, 29, 32, 33]. Direct comparative data on seizure risk across parenteral antipsychotic formulations in patients with structural brain disease are lacking.

This has practical implications. Haloperidol, despite being a first-generation antipsychotic, is often used parenterally in inpatient and delirium settings because of familiarity, rapid onset, and relatively low seizure liability compared with low-potency phenothiazines [32, 36, 40, 41]. Among second-generation agents, short-acting intramuscular formulations of olanzapine and ziprasidone have been used for acute agitation, and available evidence from psychiatric emergency care suggests that these agents can be effective in selected patients [40, 41]. From a seizure-risk standpoint, ziprasidone is generally viewed as a lower-risk second-generation option, whereas olanzapine likely warrants more caution because its broader receptor-binding profile, including greater histaminergic and muscarinic activity, places it closer to the intermediate range of seizure liability [33, 37, 39].

Intravenous antipsychotic use requires separate caution. In contemporary practice, antipsychotics used for rapid tranquilization are generally administered intramuscularly rather than intravenously, in part because intravenous administration has been constrained by cardiac safety concerns, particularly QT prolongation and torsades de pointes risk with agents such as haloperidol and droperidol [40, 41]. Thus, in hospitalized brain tumor patients, an intravenous antipsychotic should not be viewed as a safer neurologic option simply because it permits rapid delivery. The route may add cardiopulmonary monitoring requirements without reducing seizure vulnerability, which continues to depend principally on agent selection, dose, titration, and underlying cortical instability.

For neuro-oncology inpatients, parenteral antipsychotic use should therefore follow the same seizure-informed logic as oral treatment, but with even greater attention to reversible precipitants and time-sensitive co-management. When urgent behavioral control is necessary, lower-risk agents or lower-risk formulations should be favored when feasible; rapid dose escalation and antipsychotic polypharmacy should be avoided; benzodiazepine coadministration should be used thoughtfully, especially when nonconvulsive seizures, respiratory compromise, or oversedation are concerns; and the need for concurrent anti-seizure medication review should be reassessed early. In practice, this means that haloperidol may remain reasonable for selected hospitalized patients when used conservatively, intramuscular ziprasidone may be attractive when a lower-risk second-generation option is desired, olanzapine requires greater caution [24, 29, 33, 35–37, 40, 41].

## Antipsychotic Use in Brain Tumor Patients

### Amplified and Context-Dependent Risk

Antipsychotic prescribing in patients with brain tumors occurs in the context of a markedly heightened seizure susceptibility. Tumor-related cortical irritation, network disconnection, peritumoral edema, metabolic instability, and therapy-induced neurotoxicity collectively reduce the seizure threshold long before any psychotropic agent is introduced [18, 20]. See Table 2 for categories and features of various seizure risk modifiers in brain-tumor patients receiving antipsychotics. In this context, the seizure risk associated with antipsychotics is amplified not only by their intrinsic proconvulsant potential, but also by the convergence of tumor biology, psychiatric activation, antiseizure medication effects, and acute medical stressors.

**Table 2:** Seizure risk modifiers relevant to antipsychotic prescribing in patients with brain tumors

Domain	Modifier	Potential effect on seizure threshold	Clinical clues	Implication for antipsychotic prescribing
Tumor biology [1, 5, 11, 14, 17, 18, 20]	Low-grade glioma/glioneuronal tumor	Chronic cortical remodeling and persistent peritumoral hyperexcitability	Longstanding seizures, seizure-predominant presentation	Prefer lower-risk agents; anticipate high baseline vulnerability
	High-grade glioma	Necrosis, hemorrhage, edema, acute injury to inhibitory circuits	Rapid neurologic decline, mass effect, worsening edema	Avoid rapid escalation during unstable periods
	IDH-mutant glioma	Tumor-associated epileptogenicity and high preoperative seizure burden	Diffuse glioma with early seizures	Lower threshold for seizure-informed psychopharmacology
Tumor location [2, 5, 13, 14, 17–19, 27]	Cortical involvement	Direct disruption of excitatory-inhibitory balance in seizure-prone cortex	Cortically based tumors or metastases	Treat as higher baseline seizure-risk state
	Frontal/temporal lobe involvement	Regions are both highly epileptogenic and strongly linked to agitation, psychosis, or disinhibition	Hallucinations, paranoia, impulsivity, affective lability	High likelihood of needing antipsychotic treatment, but with greater caution
Peritumoral state [1, 5, 17, 18, 20, 27]	Edema	Cortical irritation and impaired network stability	Steroid-responsive symptoms, MRI edema burden	Start low, titrate slowly, reassess after edema treatment
	Hemorrhage/necrosis	Acute tissue injury and inflammatory destabilization	Sudden worsening, focal deficits, acute mental-status change	Reassess neurologic context before attributing change to primary psychiatric illness
Treatment-related [5, 14, 17, 18, 20, 21, 27]	Postoperative state	Tissue manipulation, transient edema, metabolic shifts	Early postoperative delirium or agitation	Use conservative dosing; consider neurology co-management
	Radiation-related injury/necrosis	Delayed edema, inflammation, white-matter injury	New confusion, seizures, cognitive slowing after radiotherapy	Reevaluate before antipsychotic escalation
	Corticosteroids	Psychiatric activation, sleep disruption, metabolic perturbation	Steroid-induced insomnia, mania, psychosis	Correct contributors where possible before escalating antipsychotics
ASM-related [1, 15, 21–25]	Levetiracetam	Behavioral adverse effects may increase apparent need for antipsychotic treatment	Irritability, agitation, anxiety, aggression	Reconsider ASM choice before intensifying antipsychotics
	Enzyme-inducing ASMs	May lower antipsychotic concentrations and complicate efficacy	Carbamazepine, phenytoin	Watch for underdosing, false nonresponse, or compensatory escalation
	ASM polytherapy	Additive cognitive, sedative, and neurobehavioral burden	Fatigue, slowed thinking, imbalance, delirium	Simplify regimens when possible; avoid layering sedating psychotropics
Systemic/metabolic [17, 21, 27]	Electrolyte, glucose, infectious, or hypoxic stressors	Common precipitants of both seizures and delirium	Hyponatremia, infection, hypoxia, hyper/hypoglycemia	Correct reversible drivers before changing antipsychotics
	Sleep deprivation	Lowers seizure threshold and worsens agitation	ICU care, steroid use, pain, hospitalization	Prioritize sleep restoration and environmental stabilization
Psychiatric syndrome [2, 21, 27]	Delirium/agitation/psychosis	Increases urgency for treatment but often coexists with reversible neurologic or metabolic triggers	Pulling lines, severe distress, hallucinations, aggression	Treat symptoms, but search aggressively for causes
Prescribing-related [24, 25, 29, 32, 33]	Rapid titration/high dose	Exaggerates destabilization in a vulnerable brain	Escalation over hours to 1–2 days	“Start low, go slow” is especially important in BTRE
	Antipsychotic polypharmacy	Increases cumulative receptor burden and adverse-effect load	Multiple scheduled agents or PRN stacking	Avoid whenever possible

Abbreviations: *ASM*, antiseizure medication; *BTRE*, brain tumor-related epilepsy; *IDH*, isocitrate dehydrogenase; *MRI*, magnetic resonance imaging.

Seizure risk in brain tumor patients is context−dependent and reflects the combined effect of tumor biology, cortical involvement, treatment−related instability, ASM burden, systemic stressors, and prescribing strategy rather than antipsychotic choice alone [1, 5, 14, 15, 17–27, 42, 43]

### Hyperexcitability, ASM Burden, and the Bidirectional Effects of Antipsychotics

Brain tumor–related epilepsy contributes to cognitive impairment, emotional dysregulation, slowed processing speed, and executive dysfunction beyond the tumor itself. Interictal epileptiform discharges, recurrent seizures, and chronic antiseizure medication exposure may further compound this neuropsychological burden [1].

Under these conditions, rapid titration, higher doses, or polypharmacy may further destabilize already compromised networks. In addition, commonly used antiseizure medications may worsen behavioral symptoms. Levetiracetam prophylaxis in seizure-naïve brain tumor patients has been associated with higher rates of agitation, irritability, anxiety, depression, and behavioral dysregulation than its use in established BTRE, potentially increasing the likelihood of antipsychotic exposure in an already vulnerable population [15]. Levetiracetam should be avoided due to its high behavioral adverse-effect burden (7–13%), particularly in this population [21].

Thus, antipsychotic decision-making in brain tumor patients cannot be disentangled from ASM selection, tumor location, and the evolving neurophysiologic milieu.

### Lack of Direct Evidence and Practical Implications

Controlled studies directly evaluating seizure risk from antipsychotics in patients with brain tumors are lacking. Available guidance therefore relies on pathophysiologic inference and evidence from epilepsy, psychopharmacology, and neuro-oncology. In practice, risk is likely amplified by common clinical precipitants, including postoperative cortical irritation, radiation necrosis, tumor-associated edema, hyponatremia, infection, hypoxia, and polypharmacy with other proconvulsant agents [18, 20].

Accordingly, antipsychotic selection should favor lower-risk agents, when possible, with aripiprazole and risperidone generally preferred, whereas clozapine should be reserved for refractory cases and used only with slow titration, close monitoring, and concurrent consideration of antiseizure management. These principles underscore the need for close collaboration among psychiatry, neurology, and neuro-oncology when initiating or titrating antipsychotics in brain tumor patients.

### Clinical Decision Framework for Antipsychotic Use in BTRE

#### Step 1. Define the Syndrome

Clarify whether the target symptom is agitation, psychosis, delirium, mood dysregulation, or mixed behavioral disturbance, and consider whether it is tumor-related, BTRE-related, ASM-related, steroid-induced, metabolic, or multifactorial.

#### Step 2. Exclude Reversible Contributors

Before escalating antipsychotic treatment, evaluate for tumor progression, edema, infection, metabolic disturbance, steroid effects, and ASM toxicity. In many patients, correction of these factors reduces both seizure risk and behavioral symptoms.

#### Step 3. Estimate Baseline Seizure Vulnerability

Assess for high-risk features, including cortical, frontal, or temporal lobe tumors; low-grade gliomas, glioneuronal tumors, and cortically based metastases; prior seizures or BTRE; peritumoral edema, cortical irritation, postoperative state, radiation necrosis, and metabolic instability. These factors determine whether the patient is likely to tolerate antipsychotic initiation or whether seizure-risk mitigation is needed first.

#### Step 4. Review the ASM Regimen

Antipsychotic safety cannot be separated from ASM choice. Levetiracetam should be reconsidered when irritability, agitation, anxiety, depression, or psychosis are prominent, given its behavioral adverse-effect burden in brain tumor patients. When psychiatric symptoms are substantial, alternatives such as brivaracetam, lacosamide, valproate, or lamotrigine may be better tolerated, depending on tumor type, metabolic context, and affective comorbidity. Potential drug-drug interactions with enzyme-inducing or enzyme-inhibiting ASMs should also be reviewed before psychotropic changes.

#### Step 5. Select the Antipsychotic by Seizure-risk Hierarchy

Agent selection should follow a seizure-informed hierarchy. Aripiprazole and risperidone are generally preferred first-line because of their lower seizure liability. Olanzapine and quetiapine may be used when clinically necessary but require greater caution. Clozapine should be reserved for refractory cases and used only with slow titration, close monitoring, and active ASM co-management.

#### Step 6. Dose Conservatively

Start low and titrate slowly, especially during postoperative periods, radiation-related edema, steroid initiation or taper, or metabolic instability. Avoid abrupt dose escalation and avoid antipsychotic polypharmacy whenever possible.

#### Step 7. Determine Whether ASM Co-management is Needed

Routine prophylaxis in seizure-naïve patients is not generally recommended [42, 43], but targeted ASM co-management is appropriate in patients with prior seizures, cortical or temporal lobe tumors, radiation necrosis or substantial edema, postoperative delirium requiring higher antipsychotic exposure, or clozapine use.

#### Step 8. Monitor for Early Warning Signs

Monitor for worsening agitation, irritability, depression, hallucinations, confusion, myoclonus, stuttering, speech change, new focal neurologic symptoms, and decline in attention, processing speed, or executive function. These may reflect cortical destabilization, tumor progression, ASM toxicity, metabolic disturbance, or impending seizure rather than primary psychiatric worsening.

#### Step 9. Reassess When the Neurologic Context Changes

Reevaluate the regimen after surgery, radiation, chemotherapy, steroid changes, ASM titration, fluctuating edema, metabolic disturbance, or any new cognitive or behavioral decline.

#### Step 10. Maintain Interdisciplinary Oversight

Safe prescribing depends on coordination among psychiatry, neurology, and neuro-oncology, particularly when seizures are active, tumors are cortically based, edema is fluctuating, or ASM or steroid adjustments are anticipated.

#### Implementation in Interdisciplinary Care

Caregivers, nursing staff, and allied health clinicians are often the first to detect subtle behavioral change. New agitation, emotional lability, hallucinations, myoclonus, speech change, or cognitive decline may signal cortical destabilization, tumor progression, ASM toxicity, metabolic derangement, or impending seizure rather than primary psychiatric worsening alone. Early recognition can prevent unnecessary antipsychotic escalation and prompt timely correction of reversible contributors.

#### Integrative Principle

Antipsychotic risk in neuro-oncology is context-dependent, shaped by tumor biology, BTRE, ASM burden, metabolic state, and ongoing oncologic treatment rather than by drug properties alone. Safe prescribing therefore requires a deliberate, adaptive, seizure-informed framework embedded in interdisciplinary care.

## Future Directions

Future work should address the absence of controlled data on antipsychotic-associated seizure risk in patients with brain tumors, a major gap that leaves neuro-oncology prescribing reliant on extrapolation despite parallel evidence on BTRE mechanisms, tumor-related network disruption, and psychotropic seizure liability. Priorities include prospective, agent-specific studies that define how known risk gradients across antipsychotics behave in the setting of tumor-related hyperexcitability; predictive models integrating tumor histology, cortical involvement, peritumoral excitability, seizure burden, interictal epileptiform activity, ASM tolerability, lesion-linked psychiatric syndromes, and psychotropic titration strategies; and interventional trials clarifying when selective ASM co-management may be warranted, particularly with higher-risk antipsychotics, postoperative delirium, corticosteroid-induced psychosis, or levetiracetam-related behavioral toxicity. Systems-level advances should also evaluate embedded consultation-liaison psychiatry within neuro-oncology, standardized neurology-psychiatry risk assessment before antipsychotic initiation, routine cognitive, behavioral, and seizure monitoring, and clinician training to recognize early markers of cortical instability such as myoclonus, stuttering, agitation, and neurocognitive decline. Collectively, these directions support a precision psychiatry paradigm in which treatment is informed by tumor biology, circuitry disruption, ASM interactions, and the evolving neurophysiologic state of the tumor-bearing brain.

## Conclusion

Psychiatric and behavioral disturbances in patients with brain tumors arise within a uniquely vulnerable neurobiological environment shaped by tumor location, network disruption, peritumoral hyperexcitability, treatment-related neurotoxicity, metabolic instability, and the cognitive burden of BTRE. Although the seizure liability of antipsychotics is well described in general psychopharmacology, those data cannot be directly applied to neuro-oncology, where baseline seizure risk is far higher and vulnerability is amplified by structural brain disease, cortical infiltration, tumor-induced synaptic remodeling, ASM-related behavioral toxicity, and fluctuating systemic precipitants. Antipsychotic prescribing in this setting therefore requires a context-dependent, seizure-informed strategy that weighs tumor-induced cortical hyperexcitability, ASM-related neuropsychiatric destabilization, high baseline psychiatric and cognitive burden, and reversible metabolic or medical triggers. In practice, this means prioritizing lower-risk agents such as aripiprazole and risperidone when feasible, reserving olanzapine or quetiapine for selected situations and clozapine for refractory cases with ASM co-management, using slow titration and avoiding rapid dose escalation, reassessing edema, steroid effects, infection, sleep disruption, and metabolic abnormalities, optimizing ASM selection including avoidance of levetiracetam in psychiatric presentations when appropriate, and aligning decisions through close psychiatry-neurology-oncology collaboration. Safe and effective antipsychotic use in neuro-oncology is achievable, but only through an interdisciplinary precision psychiatry framework tailored to the biologic and clinical instability of the tumor-bearing brain.

## Key References

- Avila EK, Tobochnik S, Inati SK, et al. Brain tumor-related epilepsy management: A Society for Neuro-oncology (SNO) consensus review on current management. Neuro Oncol. 2024;26(1):7–24. doi: 10.1093/neuonc/noad154
  - ○ This recent SNO consensus review is central to the article’s framing of brain tumor-related epilepsy as a biologically complex and clinically dynamic condition. It supports the discussion of tumor-associated hyperexcitability, seizure vulnerability, and the need for individualized management in neuro-oncology.
- Maschio M, Perversi F, Maialetti A. Brain tumor-related epilepsy: an overview on neuropsychological, behavioral, and quality of life issues and assessment methodology. Front Neurol. 2024;15:1480900. doi: 10.3389/fneur.2024.1480900
  - ○ This article is important because it expands BTRE beyond seizure frequency alone and emphasizes neuropsychological, behavioral, and quality-of-life consequences. Its concept of “BTRE-induced disability” directly supports the manuscript’s argument that antipsychotic prescribing must account for cognition, behavior, seizure risk, and overall functioning.
- Dono F, Consoli S, Evangelista G, et al. Levetiracetam Prophylaxis Therapy for Brain Tumor-Related Epilepsy (BTRE) Is Associated With a Higher Psychiatric Burden. Front Neurol. 2021;12:806839. doi: 10.3389/fneur.2021.806839
  - ○ This study is clinically relevant because it highlights the psychiatric adverse-effect burden associated with levetiracetam prophylaxis in seizure-naïve brain tumor patients. It supports the manuscript’s emphasis that antiseizure medication selection can influence behavioral stability and may affect the need for antipsychotic treatment.
- Reichelt L, Efthimiou O, Leucht S, Schneider-Thoma J. Second-generation antipsychotics and seizures - a systematic review and meta-analysis of serious adverse events in randomized controlled trials. Eur Neuropsychopharmacol. 2023;68:33–46. doi: 10.1016/j.euroneuro.2022.12.010
  - ○ This meta-analysis provides the key general-population comparator for second-generation antipsychotic seizure risk. It is important because it shows low intrinsic seizure liability in randomized psychiatric trials while also underscoring the limitation that such data may not directly apply to patients with structural brain disease.
- Newton HB, Wojkowski J. Antiepileptic Strategies for Patients with Primary and Metastatic Brain Tumors. Curr Treat Options Oncol. 2024;25(3):389–403. doi: 10.1007/s11864-024-01182-8
  - ○ This review is useful for the manuscript’s practical recommendations because it addresses antiseizure medication strategies in patients with primary and metastatic brain tumors. It supports the discussion of ASM selection, tolerability, and the importance of minimizing psychiatric and drug-interaction burden in neuro-oncology care.

## Data Availability

No datasets were generated or analysed during the current study.
